# Familiar Tonal Context Improves Accuracy of Pitch Interval Perception

**DOI:** 10.3389/fpsyg.2017.01753

**Published:** 2017-10-09

**Authors:** Jackson E. Graves, Andrew J. Oxenham

**Affiliations:** Department of Psychology, University of Minnesota, Minneapolis, MN, United States

**Keywords:** expectation, discrimination, harmony, melody, tonality

## Abstract

A fundamental feature of everyday music perception is sensitivity to familiar tonal structures such as musical keys. Many studies have suggested that a tonal context can enhance the perception and representation of pitch. Most of these studies have measured response time, which may reflect expectancy as opposed to perceptual accuracy. We instead used a performance-based measure, comparing participants’ ability to discriminate between a “small, in-tune” interval and a “large, mistuned” interval in conditions that involved familiar tonal relations (diatonic, or major, scale notes), unfamiliar tonal relations (whole-tone or mistuned-diatonic scale notes), repetition of a single pitch, or no tonal context. The context was established with a brief sequence of tones in Experiment 1 (melodic context), and a cadence-like two-chord progression in Experiment 2 (harmonic context). In both experiments, performance significantly differed across the context conditions, with a diatonic context providing a significant advantage over no context; however, no correlation with years of musical training was observed. The diatonic tonal context also provided an advantage over the whole-tone scale context condition in Experiment 1 (melodic context), and over the mistuned scale or repetition context conditions in Experiment 2 (harmonic context). However, the relatively small benefit to performance suggests that the main advantage of tonal context may be priming of expected stimuli, rather than enhanced accuracy of pitch interval representation.

## Introduction

Pitch, a primary dimension of auditory sensation, is an attribute closely related to the fundamental frequency (F0) or overall periodicity of a sound. In speech, rising and falling pitch contours serve as cues to a speaker’s emotions, intentions, and emphasis, and as cues to semantic meaning in tonal languages. In music, sequences of pitch define melody and simultaneous combinations of pitch define the harmony of chords. In Western music, as in many other traditions, pitches are organized into discrete categories within a tonal hierarchy such as a musical key. Listeners, especially those with musical training, are sensitive to these hierarchies, rating some notes as better “completions” than others following a musical scale (Krumhansl and Shepard, 1979), or following a single chord or sequence of chords (Krumhansl and Kessler, 1982). The resulting “tone profiles” of perceived pitch relationships within the key cannot be predicted simply from proximal stimulus similarities, and instead are thought to reflect prior knowledge and exposure (Parncutt and Bregman, 2000). Tonal structure is a strong factor influencing psychological expectancies for both melody and harmony (Schmuckler, 1989). For melodies, listener expectations are also heavily influenced by contour (Cuddy and Lunney, 1995), in accordance with contour-based models (Narmour, 1990). Thus, to fully describe listener expectations for melodic continuation, it is necessary to consider both tonal structure and melodic contour as separate influences (Graves et al., 2014).

Sensitivity to tonal hierarchies may be the result of a process of statistical learning, wherein listeners come to expect musical patterns to which they have been frequently exposed. Statistical learning for pitch patterns has been observed on a small scale in both infants and adults (Saffran et al., 1999), in a process analogous to learning of word segmentation in language development. On a larger scale, tonal expectations in Western listeners are well explained by statistical regularities in familiar Western music such as folk songs and chorales (Pearce and Wiggins, 2006). This learning likely occurs very early in life, as infants as young as 7 months are sensitive to familiar tonal structures (Cohen et al., 1987). However, specialization for a particular tonal hierarchy may take time to develop fully: while 6–8 month-old infants are equally able to detect violations of various tonal structures, Western adults are most sensitive to violations of the Western diatonic scale (Lynch et al., 1990; Trainor and Trehub, 1992).

Once learned, tonal sensitivity is a robust phenomenon. Familiar melodies are stored in long-term memory based on tonal structure, not only contour (Dowling and Fujitani, 1971), and even short-term memory for novel melodies is influenced by tonality (Dowling, 1978; Boltz, 1991). In fact, for musically trained listeners, tonal hierarchies need not even be cued physically: tone profiles of pitch relationships within a musical key can also be measured following imagined (not physically presented) tonal hierarchies (Vuvan and Schmuckler, 2011).

Accessing these overlearned tonal hierarchies can facilitate pitch processing when the relevant pitches are highly expected within the tonal structure. For various pitch processing tasks, response times are faster for expected than for unexpected chords, based on the preceding harmonic progressions (Bharucha, 1987; Bigand and Pineau, 1997; Tillmann et al., 2008; Tillmann and Marmel, 2013), as well as for notes primed by melodic context (Marmel et al., 2008, 2011). The mechanism of this facilitation of processing may be either priming of expected pitches, or enhanced perception and representation of important pitches or harmonies within the tonal hierarchy. Under the former expectation-based explanation, a pitch that is predicted or expected by a tonal hierarchy may produce a faster response time simply because less time is required to react to an unsurprising or predictable event. This explanation is favored by most reaction-time studies, e.g., Bigand and Pineau (1997). Under the second perceptual-accuracy-based explanation, however, response times could be faster with tonal context because the representation of pitch at some level in the auditory system becomes more accurate, rendering the task easier. This may take the form of anticipatory activation of expected pitches (e.g., Bharucha, 1987). Increased event-related potential (ERP) amplitudes to pitches high in the tonal hierarchy provide additional evidence for enhanced neural representation of these pitches (e.g., Krohn et al., 2007).

If the decreased response times in these studies reflect an enhanced sensory representation of pitch, we might expect to observe improvements in measures of performance or accuracy as well. One such measure is pitch discrimination, where the listener directly compares two pitches presented in sequence. For this task, tonal context has been found to improve accuracy, but the observed effects have been small relative to effects on response time, and in some cases may be modulated by differences in timbre between tones (Warrier and Zatorre, 2002; Marmel et al., 2008; Borchert and Oxenham, 2010). Harmonic priming studies have used a dissonance detection task in which the listener must detect the presence of an augmented root or augmented fifth (both highly dissonant chord members in Western music). Tonal context also has small and inconsistent effects on accuracy for this task (Bigand and Pineau, 1997; Tillmann and Marmel, 2013), in contrast with robust effects on response time. However, in these tasks, the mistuning can be detected also by the presence of acoustics beats in the waveform of the dissonant interval (McDermott et al., 2010b), meaning that the pitch interval itself need not be discriminated by the participants. Thus, the lack of a robust effect of tonal context on task accuracy in these situations does not necessarily imply a lack of pitch enhancement through tonal context.

It is possible that tonal context effects are stronger for pitch *interval* discrimination than for simple pitch discrimination. Pitch intervals determine tonal hierarchies and set pitch apart from other auditory dimensions such as brightness and loudness (McDermott et al., 2008; Graves et al., 2014). Interval discrimination may be a more difficult task, due to the higher cognitive load required to represent distances (intervals) as opposed to individual values (pitches) in working memory. This could be the reason that discrimination thresholds, or difference limens (DLs), for pitch intervals are large compared to basic pitch DLs, which are exceptionally low among auditory dimensions (McDermott et al., 2010a). With more room for improvement, one might expect that any enhancement of the sensory representation of pitch would be especially beneficial on a pitch interval perception task. In addition, one known effect of tonal structure on pitch interval perception is that tonality allows for categorical perception of discrete intervals, as opposed to a continuous range. There is some evidence that musicians may more accurately discriminate pitch intervals at category boundaries than within an interval category, although this effect is not robust, and is sensitive to differences in experimental methodology (Burns and Ward, 1978). However, the effect was not observed at all in non-musicians, suggesting that categorical perception, if present, is learned. In a convergent finding, small frequency oscillations are more easily detected when centered around perfect octaves and fifths than neighboring intervals (Demany and Semal, 1992). The subjective “octave” category is slightly stretched relative to a physical octave (doubling in frequency), but approaches the physical octave when tonal context is introduced (Cuddy and Dobbins, 1988). Activating a tonal hierarchy could potentially enhance pitch interval perception by sharpening distinctions between primed interval categories. In other words, within a tonal context, a musical interval that is larger or smaller than expected may result in the second note being perceived as a “sour note” with respect to its expected pitch value, rather than in terms of the interval size between it and the preceding note.

A previous study found that the discrimination of musical intervals was better following a short melody than for intervals presented in isolation, suggesting that tonal context does enhance perception of pitch intervals (Wapnick et al., 1982). However, certain aspects of that study’s methodology leave its results open to interpretation. Firstly, only participants with a very high degree of musical experience were tested, and these participants received additional extensive training on an interval labeling task before completing the interval discrimination task. Many of them reported having absolute pitch, and all of them showed some degree of absolute pitch labeling ability, making it unclear whether the participants even needed to compare the two tones in each trial to complete the task. Although benefit from melodic context should not depend on absolute pitch possession, this may have transformed the putative relative-pitch task into functionally an absolute-pitch task. Secondly, the first pitch of the first interval on discrimination trials was always held constant, potentially allowing participants to use absolute pitch, instead of relative pitch, and so employ basic pitch discrimination instead of pitch interval discrimination. Thirdly, no distinction was made between a musical context that defines a congruent tonal hierarchy (such as a major key) and a tonally incongruent musical context: participants heard either a familiar melody or nothing. Thus, the benefit of a tonal context may be due to the reinforcement of tonality, or simply due to the presence of any context pitches, regardless of their tonal congruence.

The present study sought to determine whether a prior tonal context enhances pitch representations in a way that improves pitch interval discrimination. In order to ensure that participants were perceiving relative pitch intervals, we roved all absolute pitches in the study across a continuous range of fundamental frequencies. To dissociate various potential interpretations of a difference between familiar melodic context and no context, we also included three control conditions: a Repetition condition to test the effect of simply reinforcing the target pitch without any reference to a tonal (e.g., major or minor) center, and two unfamiliar melodic contexts (Mistuned and Whole-Tone Scales) for comparison with the more familiar (diatonic, Major Scale) context. If familiar tonal hierarchies do in fact facilitate pitch processing by enhancing the sensory representation of pitch or pitch intervals, we would predict that tonal context improves performance in an interval discrimination task, but only in cases in which the context provides congruent tonal cues. The first experiment established context using a melodic sequence of single pitches. The second experiment established context using a harmonic sequence of multiple pitches in the form of an authentic cadence. In all cases, care was taken to ensure that none of the context tones was of the same pitch class as the test tone itself, to avoid the possibility that participants were making a direct comparison between the test tone and one of the context tones.

## Experiment 1: Melodic Context for Pitch Intervals

### Materials and Methods

#### Stimuli

Participants heard sequences of pitches carried by harmonic complex tones. The tones were generated with all harmonics lower than the Nyquist frequency (22.05 kHz), and were lowpass filtered with a cutoff frequency of 200 Hz and a -12 dB/octave slope. The overall level of each tone after filtering was 60 dB SPL. The tones were generated within MATLAB (The Mathworks, Natick, MA, United States), using a 24-bit L22 soundcard (Lynx Studio Technology, Costa Mesa, CA, United States), presented diotically through HD650 headphones (Sennheiser United States, Old Lyme, CT, United States) at a sampling rate of 44.1 kHz.

**FIGURE 1:**
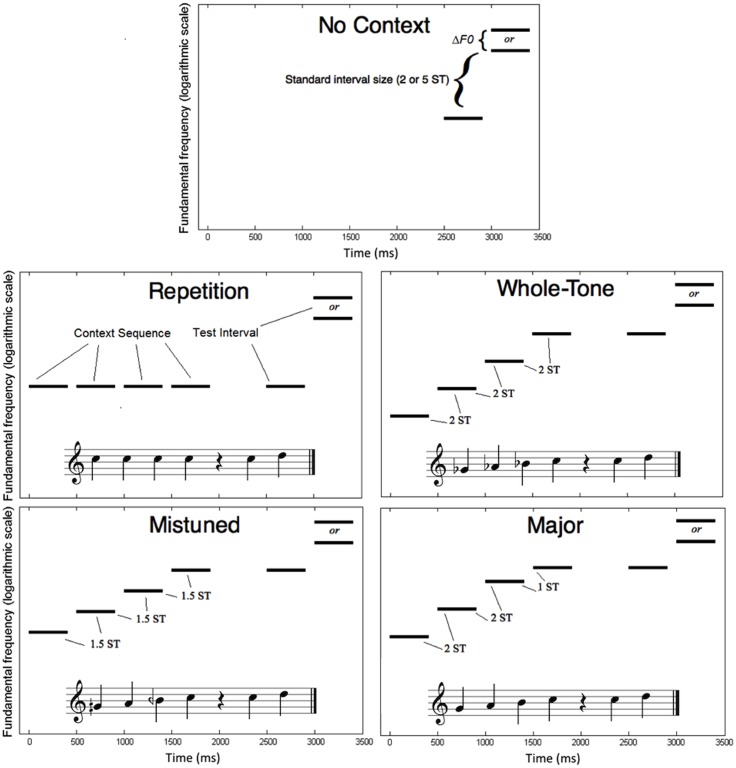
Schematic diagrams in spectrogram form of a single trial in each of the five conditions for Experiment 1 (melodic context). Pitch distances are labeled in semitones (ST). Context conditions are also illustrated with musical notation.

**Figure 1** shows the paradigm for stimulus presentation in the five melodic context conditions. The task-relevant stimuli on each trial were two tones presented sequentially, each with a duration of 400 ms, including 10-ms raised-cosine rise and fall ramps, separated by a gap of 100 ms. Trials in the No Context condition consisted only of the test interval formed by these two test tones. The F0 of the first test tone was randomly chosen from a uniform distribution within a 1.5-octave range from 200 to 565.69 Hz (approximately G3 to C#5). On half of the trials, the second test tone’s F0 was higher than that of the first by a ratio exactly equal to a standard interval in the diatonic equal-tempered scale. On the other half of trials, the second test tone’s F0 was higher than the frequency that would be chosen by the standard interval size; we termed the ratio of this discrepancy Δ*F0*. Thus, if the test tone was one semitone higher than the frequency that would have been selected in the standard interval, Δ*F0* would be approximately 6% (2^1/12^). Participants were instructed to judge whether each interval was “small, in tune” (when the F0 difference was exactly the standard interval size) or “large, mistuned” (when the F0 difference was greater than the standard interval size by Δ*F0*). In this way, participants had the option of using either the size cue (“small” or “large”) or the tuning cue (“in tune” or “mistuned”) to complete this task. Two standard interval sizes were tested in two separate phases of the experiment. These were two semitones (a major second) or five semitones (a perfect fourth) in the equal-temperament tuning system. We chose common intervals in Western tonal music because our participants were more likely to have been exposed to Western tonal music than other musical styles. By avoiding standard intervals larger than five semitones, we avoided repeating any pitch classes from the context sequence.

On trials in the other four conditions, the test interval was preceded by a melodic context sequence, consisting of four tones with durations of 400 ms each (including 10-ms raised-cosine rise and fall ramps), separated by 100-ms gaps, with 600 ms silence between the context sequence and the final two test tones. In each context condition, the F0 of the final tone in the context sequence was equal to the F0 of the first tone in the test interval. In the Repetition condition, all four context tones had the same F0 as the first test tone. In the Mistuned condition, each tone in the context sequence was exactly 1.5 semitones higher than the previous tone. In the Whole Tone condition, each tone in the context sequence was exactly two semitones higher than the previous tone. Finally, in the Major condition, the context sequence corresponded to a major (diatonic) scale ascending from the dominant (5th) scale degree to the tonic, with successive interval sizes of two semitones, two semitones, and one semitone. These four context conditions were designed to dissociate the effects of pitch reinforcement, directional context, tuning cues, and tonal hierarchy, respectively. All four context conditions provide some pitch reinforcement: additional examples of the first pitch of the test interval may be helpful. Mistuned, Whole Tone, and Major conditions all provide directional context: upward intervals of fixed size are presented, against which the test interval could be compared. Only Whole Tone and Major conditions fit within the Western 12-tone chromatic scale, and only the Major condition fits within the Western hierarchical diatonic scale.

#### Participants

Twenty-one participants, 9 male and 12 female, were recruited from the Twin Cities campus of the University of Minnesota. They ranged from 18 to 25 years of age (*Mean* = 19.8, *SD* = 1.9), and from 0 to 15 reported years of musical experience (*Mean* = 5.9, *SD* = 5.2), with musical experience defined as regularly playing any musical instrument. All participants were screened for normal audiometric hearing thresholds, defined as not exceeding 20 dB hearing level (HL) for frequencies between 250 and 8000 Hz. All protocols were approved by the University of Minnesota Institutional Review Board. The experiment was completed in a single 2-h session per participant. All participants provided written informed consent, in accordance with the Declaration of Helsinki, and were compensated for their participation.

#### Procedure

To allow the participants to gain familiarity with the standard interval size (two or five semitones), each participant completed the entire experiment for one standard interval before being tested on the other standard interval. The order of the standard interval presentation was counterbalanced between participants, such that 11 participants completed the procedure for the two-semitone standard first, while 10 participants completed the procedure for the five-semitone standard first.

Because the task was novel and not intuitive for many participants, each participant began with orientation and training before moving on to the testing phase. The orientation phase consisted of listening to five labeled examples of the small, in-tune interval and five examples of the large, mistuned interval. For this demonstration, the Δ*F0* ratio was fixed at 8% (larger than a semitone). During the orientation phase, participants did not respond, but merely listened to the labeled examples.

The training phase consisted of 3 blocks of 40 trials each in the No Context condition. For the first block, Δ*F0* was fixed at 12.6% (just larger than two semitones). For the second block, Δ*F0* was 8%, and for the third it was 5% (just under a semitone). No time limit was imposed on responses during this training period. Participants generally performed near ceiling during this training phase, making few errors, as the Δ*F0*s used were large.

Following training, each participant’s DL for Δ*F0* in the No Context condition was estimated in a pilot phase of the experiment using an adaptive tracking procedure. The geometric mean estimated DL was 3.1% for the two-semitone standard, 95% CI [2.3% 4.2%], and 2.8% for the five-semitone standard, 95% CI [2.0% 4.0%]. This wide range of thresholds is typical for frequency discrimination tasks, as recently illustrated in a study of 100 participants with normal hearing (Whiteford and Oxenham, 2015).

The estimated DLs, determined for each participant individually, were used to set the Δ*F0* in the main testing phase of the experiment. Based on pilot testing, participants were expected to perform at sensitive levels (below ceiling and above chance) when tested with Δ*F0* set to roughly 1/4 the threshold estimated by the adaptive tracking procedure. This discrepancy may be due to learning occurring over the course of the experiment. Accordingly, each participant was tested with Δ*F0* set to 25% of his or her initially estimated threshold. Thus, Δ*F0* was constant for each participant for each standard interval size, but different across participants and standard interval sizes according to the estimated DL.

The testing phase for each standard interval condition consisted of 25 blocks of 20 trials each. Each block contained trials with one of the five context conditions. On each trial, participants were presented with the stimulus and asked “Which kind of interval – small, in-tune or large, mistuned?” Participants were required to indicate their response via key press within 1 s of stimulus offset. The time limit was introduced in order to prevent mental rehearsal of the stimulus following the presentation. If a participant failed to respond within this time limit, the experiment program recorded a response of “small, in-tune” and proceeded to the next trial. Since this was the correct response on half of the trials, running out of time gave the participant a 50% chance of being correct. Participants were instructed to avoid running out of time, and accordingly this happened rarely: the percentage of trials on which a participant ran out of time ranged from 0.2 to 6.4% (*Mean* = 1.79%, *SD* = 1.50%).

Each participant completed five blocks for each of the five context conditions during this phase. The context condition varied from block to block, with the order of context conditions determined randomly for each consecutive set of five blocks. Participants were instructed to focus only on the final two tones (the test interval) if a context sequence was present. After the testing phase was completed for one standard interval condition, the procedure was repeated in its entirety for the other standard interval condition, starting with new orientation, training, and DL estimation periods.

#### Analysis

**FIGURE 2:**
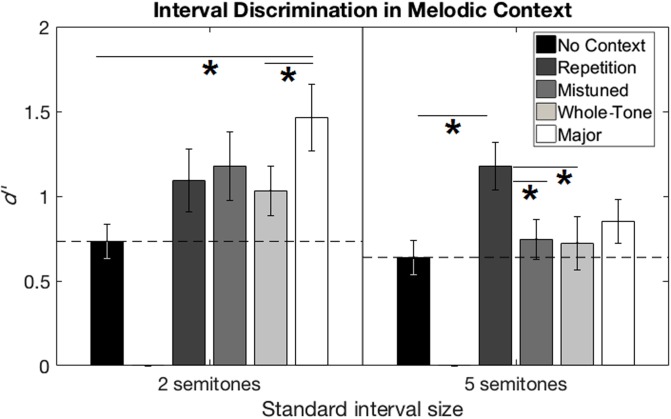
Interval discrimination performance from Experiment 1 (melodic context). Performance in *d′* is shown for the two-semitone **(left)** and five-semitone **(right)** standard interval sizes. Performance in the No Context condition was treated as a baseline (horizontal dashed line). Error bars represent ±1 standard error of the mean across participants. Horizontal solid lines with asterisks show significant pairwise comparisons between conditions for each standard interval size.

Individual participants’ sensitivity (*d′*) was estimated by subtracting the z-scored (the inverse cumulative normal distribution function) false alarm rate from the z-scored hit rate. In this calculation, a hit was defined as correctly detecting the large, mistuned interval, while a false alarm was defined as incorrectly responding “large, mistuned” when the small, in-tune interval was presented. **Figure 2** shows the pattern of performance across conditions for standard interval size. Supplementary Table S1 shows the degree of benefit for each condition relative to No Context, for each participant.

The *d′* values in the No Context condition, averaged across all participants, were between 0.5 and 1, indicating that our estimates from the pilot phase successfully produced performance that was well above chance (*d′* = 0) but below ceiling (*d′* > ∼2.5). A paired-samples *t*-test comparing *d′* values in the No Context condition for the two standard interval sizes was not significant (*p* = 0.52), suggesting that our pilot estimates of DLs in the baseline condition had been successful at targeting roughly equal levels of performance between the two standard interval sizes. Beyond that, since participants were tested at different Δ*F0* levels according to their individual estimated DLs, comparisons of absolute *d′* values between participants are uninformative. We analyzed the effect of all five context conditions with a repeated-measures ANOVA on these *d′* values, and ran *post hoc* pairwise comparisons to determine the advantage of each condition over the baseline No Context, as well as benefit of one condition over another.

### Results

The repeated-measures ANOVA on *d′* values, with two within-subjects factors of standard interval size and context condition, revealed a main effect of context condition, *F*(4,80) = 10.26, *p* < 0.001, η^2^ = 0.339. *Post hoc* pairwise comparisons of context conditions with Bonferroni correction (criterion *p* = 0.05/10 = 0.005) showed significant benefit over No Context for Major Scale (mean difference = 0.47, *p* < 0.001, Cohen’s *d* = 1.02) (Cohen, 1988) and Repetition (mean difference = 0.45, *p* < 0.001, Cohen’s *d* = 0.96) contexts, as well as an advantage of Major Scale over Whole-Tone Scale context (mean difference = 0.28, *p* = 0.001, Cohen’s *d* = 0.53). No other pairwise comparisons reached significance.

We also observed a significant interaction between context and standard interval size, *F*(4,80) = 6.049, *p* < 0.001, η^2^ = 0.232. The interaction reflects in part the difference in the benefit from the Major Scale and Repetition contexts for the two- and five-semitone standard interval sizes. We performed 25 *post hoc* pairwise comparisons to investigate this interaction: 10 comparisons between context conditions for each of the 2 standard interval sizes, and 1 comparison between standard interval sizes for each of the 5 conditions. With Bonferroni correction (criterion *p* = 0.05/25 = 0.002), for the two-semitone standard interval size, *d′* values were higher in the Major Scale context than No Context (mean difference = 0.73, *p* < 0.001, Cohen’s *d* = 1.09) or Whole Tone (mean difference = 0.43, *p* = 0.001, Cohen’s *d* = 0.90) conditions. For the five-semitone standard, *d′* values were higher in the Repetition context than No Context (mean difference = 0.54, *p* < 0.001, Cohen’s *d* = 1.04), Mistuned (mean difference = 0.43, *p* < 0.001, Cohen’s *d* = 1.28), or Whole Tone (mean difference = 0.45, *p* = 0.001, Cohen’s *d* = 0.82) conditions. No other pairwise comparisons between conditions, nor comparisons between standard intervals within conditions, reached significance. We observed no main effect of standard interval size.

### Discussion

The results of Experiment 1 suggest that performance on an interval discrimination task is significantly affected by the tonal context in which the task is performed. The Major Scale melodic context provided an advantage over the No Context or Whole-Tone Scale conditions, but no advantage over the Repetition or Mistuned-Scale conditions. Thus, no clear evidence was obtained for the benefit of establishing an over-learned (major-scale) tonal context over a simple repetition of the reference tone.

The interaction effect between context condition and standard interval size suggests that the pattern of improvement from context was different for the two- and five-semitone standard tasks. One evident difference between these patterns of results is the effect of the Repetition context and the Major Scale context in the two tasks. The best performance in the two-semitone-standard task was from the Major Scale context, whereas the best performance in the five-semitone standard task was from the Repetition context. In interpreting this difference, it is worth considering possible unintended tonal implications of the melodic context sequences. The intended interpretation of the Major Scale context was as the final four notes of an ascending major scale, ending on the tonic. Under this interpretation, both the two-semitone interval and the five-semitone interval fit in the established key. However, participants may have interpreted this sequence instead as the first four notes of an ascending major scale, beginning on the tonic. Under this interpretation, only the two-semitone interval fits in the established key. This ambiguity may explain the reduced improvement of this context sequence in the five-semitone-standard task.

The Repetition context, though intended as one level in a series of control conditions (disambiguating the effect of reiterating a reference pitch), could be interpreted as a repeating 5th scale degree (the dominant), anticipating the arrival of the tonic, which is exactly five-semitones higher. This is a common pattern in traditional Western music, and the effect may have been enhanced by the rhythmic pattern established by the temporal paradigm of this experiment, such that the final tone of the test interval can be heard to fall on a downbeat. This interpretation may explain the heightened improvement of the Repetition context sequence in the five-semitone-standard task.

If the Repetition condition had only the simple effect we intended, to reinforce the reference pitch, the simplest interpretation would be that the familiar tonal context provided an advantage over unfamiliar tonal context, but it did not provide an advantage over simple repetition of the first pitch in the test interval. This would suggest that the benefit of melodic context observed by Wapnick et al. (1982) can be disrupted with unfamiliar tonality, but may have more to do with repetition and reinforcement of target pitches than with the establishment of tonal structure. However, if we do interpret the Repetition context as inducing an accidental “tonal context” itself, these results are reasonably consistent with Wapnick et al. (1982).

## Experiment 2: Harmonic Context for Pitch Intervals

### Rationale

The results of Experiment 1 were mixed: familiar diatonic tonal context improved performance on pitch interval discrimination over no context and one unfamiliar context, Whole Tone, but not over the other unfamiliar context, Mistuned, or over simple tone repetition. Specifically for the five-semitone standard interval, familiar diatonic context provided no significant advantage over no context. One possible explanation of the small degree of benefit over no context, and the lack of benefit of the familiar tonal context with the five-semitone standard interval, is that the context of a sequence of four single tones did not establish a sufficiently strong and unambiguous sense of tonality. Indeed, many past studies have used chord progressions, rather than individual notes, to establish a clear tonality (Krumhansl and Kessler, 1982; Bharucha, 1987; Bigand and Pineau, 1997; Parncutt and Bregman, 2000; Tillmann et al., 2008). These studies have generally found stronger effects of tonality on response time than studies that used single notes (Krumhansl and Shepard, 1979; Warrier and Zatorre, 2002; Marmel et al., 2008).

To address this concern, we used chords to provide a more robust and unambiguous establishment of tonal context and to remove the potential ambiguities of the contexts used in Experiment 1. We also redefined the No Context condition in Experiment 2 to include noise bursts preceding the test interval, in order to preserve attentional and temporal cuing without pitch reinforcement.

Since musically trained listeners are more sensitive to tonal hierarchies than listeners without musical training (Krumhansl and Shepard, 1979), any effect of context may be greater in musicians than non-musicians. Indeed, for relative pitch tasks, listeners with musical experience may be uniquely sensitive to preceding context that induces tonality (Dowling, 1986). Using the results from both Experiment 1 and Experiment 2, we also investigated whether participants with musical training were more likely to see an advantage from Major Scale context.

### Materials and Methods

#### Stimuli

All pitches were carried by harmonic complex tones, generated and presented in the same manner as in Experiment 1. In the No Context condition, noise bursts were generated with overall spectral shapes similar to those of the harmonic complex tones. Specifically, the bursts consisted of band-pass noise with a center frequency of 200 Hz and shallow filter slopes of 12 dB/octave. Like the harmonic complex tones, the noise bursts had durations of 400 ms, including 10-ms raised-cosine onset and offset ramps.

**FIGURE 3:**
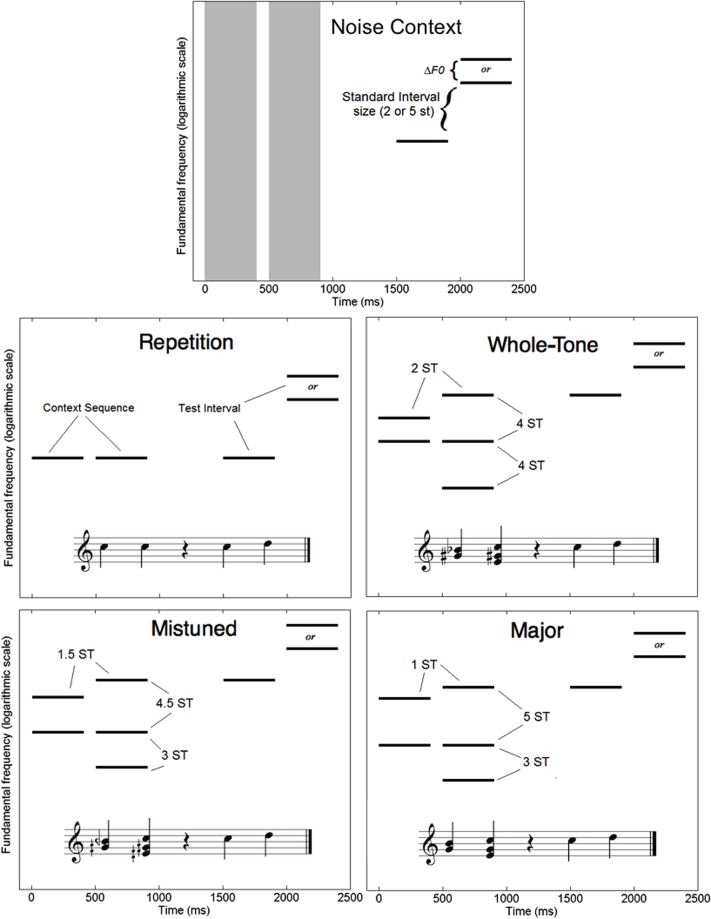
Schematic diagrams in spectrogram form of a single trial in each of the five context conditions for Experiment 2 (harmonic context). Pitch distances are labeled in semitones (ST). Gray vertical bars represent noise bursts. Context conditions are also illustrated with musical notation.

In Experiment 2, the harmonic context sequences were designed to establish a clear tonic, but without presenting the same pitch class twice, as otherwise participants could in theory compare the final tone in the test interval to a context tone one octave lower. This constraint led us to keep the context sequence very short. **Figure 3** illustrates the design for harmonic context in each of the same five conditions. In the Noise Context condition, the test interval was preceded by two noise bursts. The F0 of the first test tone was randomly chosen from trial to trial from a range of 200 to 566 Hz with uniform distribution on a logarithm scale, just as in Experiment 1. In the Repetition condition, the test interval was preceded by two context tones with the same F0 as the first test tone. In the remaining three conditions, the context sequence consisted of two simultaneous tones followed by three simultaneous tones, and the F0 of the highest of the three simultaneous tones was always equal to the F0 of the first test tone. In the Mistuned Scale condition, the pitches in the context sequence were all related by multiples of 1.5 semitones. In the Whole-Tone Scale condition, they were all related by multiples of two-semitones. In the Major Scale condition, the context sequence resembled an imperfect authentic cadence, establishing the pitch of the first test tone as tonic in a major key. It is important to note that the pitch distances are very similar between the final three conditions, although these small differences in pitch distance give rise to large differences in subjective sound quality of the resulting chords.

#### Participants

A new group of 20 participants, 6 male and 14 female, was recruited from the Twin Cities campus of the University of Minnesota. These participants ranged from 19 to 44 years of age (*Mean* = 24.4, *SD* = 6.4), and 0 to 35 years of reported musical experience (*Mean* = 6.0, *SD* = 9.0). They were all screened for normal hearing thresholds, as explained in Experiment 1. The experiment was conducted under the same IRB-approved protocol as Experiment 1, again in a single 2-h session per participant. All participants provided written informed consent and were compensated for their participation.

#### Procedure

The basic task, to discriminate between “small, in-tune” and “large, mistuned” intervals, was the same as in Experiment 1. The one-second time limit on responding was again implemented, but again was triggered only rarely: in Experiment 2, the percentage of trials on which a participant ran out of time ranged from 0.1 to 6.3% (μ = 1.25%, *SD* = 1.65%).

For similar reasons (the novelty of the task for participants), participants again completed orientation and training before the testing phase. Orientation and training for Experiment 2 were slightly modified from Experiment 1. In the orientation phase, participants heard five labeled examples of small, in-tune and five examples of large, mistuned intervals. For this demonstration, Δ*F0* was fixed at 25.1%. The increase in the demonstration value of Δ*F0* (from 8% in Experiment 1) was to ensure that participants oriented quickly to the task.

The training phase for Experiment 2 consisted of 4 blocks of 20 trials each in the Noise Context condition (test interval preceded by noise bursts). For the first block, Δ*F0* was fixed at 25.1%, for the second it was 15.8%, for the third 10%, and for the fourth 6.3%. These values of Δ*F0* are roughly in the region of one to four semitones. Once again, no time limit was imposed. As in Experiment 1, participants made few errors during this training phase, since the values of Δ*F0* were chosen to be easily discriminable, well above threshold.

An estimate of each participant’s DL was obtained by presenting them with a range of values for Δ*F0* in the Noise Context condition and choosing the lowest level at which their performance fell between 60 and 85% correct. They were then tested at exactly this level in the five context conditions. The geometric mean estimated DL was 1.5% for the two-semitone standard, 95% CI [0.9% 2.3%], and 1.2% for the five-semitone standard, 95% CI [0.9% 1.7%]. These estimated DLs are much lower than those obtained in Experiment 1, likely due to the different measurement procedure, and pilot testing indicated that they were in the sensitive region (below ceiling and above chance) for later performance in the testing phase. Thus, unlike in Experiment 1, Δ*F0* testing levels were set equal to the estimated DLs in Experiment 2, not multiplied by 25%.

As in Experiment 1, the testing phase consisted of 25 blocks of 20 trials each (five blocks per condition, presented in pseudorandom order) and the order of the standard interval procedures was counterbalanced between participants, such that 11 participants completed all conditions with the two-semitone standard first, while 9 participants completed all conditions with the procedure for the five-semitone standard first.

#### Analysis

**FIGURE 4:**
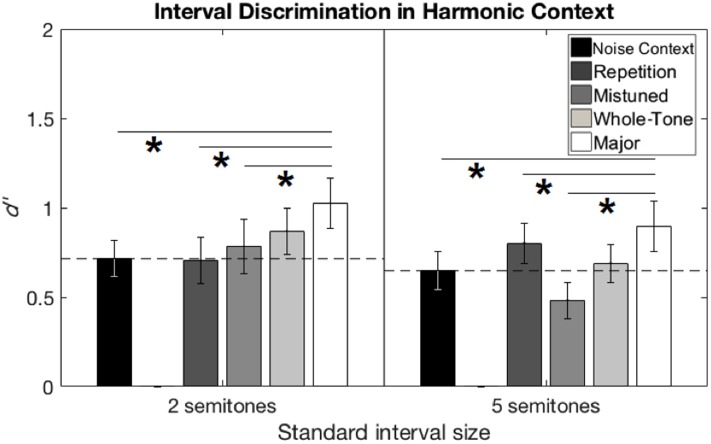
Interval discrimination performance from Experiment 2 (harmonic context). Interval discrimination performance in *d′* is shown for the two-semitone **(left)** and five-semitone **(right)** standard interval sizes. Performance in the Noise Context condition was treated as a baseline (horizontal dashed line). Error bars represent ±1 standard error of the mean across participants. Horizontal solid lines with asterisks show significant pairwise comparisons between conditions across both standard interval sizes.

The sensitivity measure *d′* was calculated for each participant, standard interval size, and context condition, based on performance in the testing phase. The value of *d′* in the Noise Context condition was used as a baseline measure, which should have been roughly equal between participants and between conditions, based on the values of F0 chosen from the pilot phase of the experiment. Indeed, a paired-samples *t*-test on these *d′* values in the Noise Context condition revealed no significant difference between standard interval sizes (*p* = 0.43). **Figure 4** summarizes performance in all conditions for both standard interval sizes. Supplementary Table S1 shows the degree of benefit for each condition relative to Noise Context, for each participant. All five context conditions were analyzed with a repeated-measures ANOVA on *d′* values.

Two further considerations can be addressed by combining results from both experiments. The first consideration involves the potential effects of learning within each experiment. To determine whether systematic improvements occurred over the course of each experiment, *d′* values for each participant were computed separately for the first block and last block of trials. A three-way repeated-measures ANOVA was performed with *d′* as the dependent variable and within-subject factors of time (first or last block), context, and standard interval size, and a between-subjects factor of experiment (1 or 2). The second consideration involves the influence of musical training. In order to evaluate the influence of musical experience on the effect of familiar tonal context, we computed the benefit of Major Scale relative to No Context or Noise Context, averaged across both standard interval sizes, for each of the 21 participants in Experiment 1 (melodic context) and the 20 participants in Experiment 2 (harmonic context). This composite measure of context effect for each individual was correlated with their ranked musical experience, using a Spearman rank-order correlation. Since participants were each tested at their individual DLs, we also correlated baseline DLs with ranked musical experience using the same method, to evaluate whether baseline performance in No Context was influenced by musical experience. For this analysis, we used DLs from Experiment 1 after multiplication by 25%, in order to represent the level at which participants were actually tested in the testing phase of both experiments.

### Results

A repeated-measures ANOVA on *d′* values, considering the within-subjects factors of standard interval size and context condition, revealed an effect of context, *F*(4,76) = 5.47, *p* = 0.001, η^2^ = 0.224, but no other main effect or interaction. Pair-wise comparisons of context conditions with Bonferroni correction (criterion *p* = 0.05/10 = 0.005) showed an advantage for Major Scale over the baseline Noise Context (mean difference = 0.28, *p* = 0.003, Cohen’s *d* = 0.59), as well as two of the other context conditions: Repetition (mean difference = 0.21, *p* = 0.003, Cohen’s *d* = 0.45) and Mistuned (mean difference = 0.33, *p* = 0.003, Cohen’s *d* = 0.65), with no other comparisons reaching significance.

For the three-way repeated-measures ANOVA including time, context, and standard interval size, the main effect of time was not significant *F*(1,39) = 0.60, *p* = 0.44, nor did any interaction involving time reach significance. Therefore, it seems that no substantial learning effects occurred over the course of the experiment.

**FIGURE 5:**
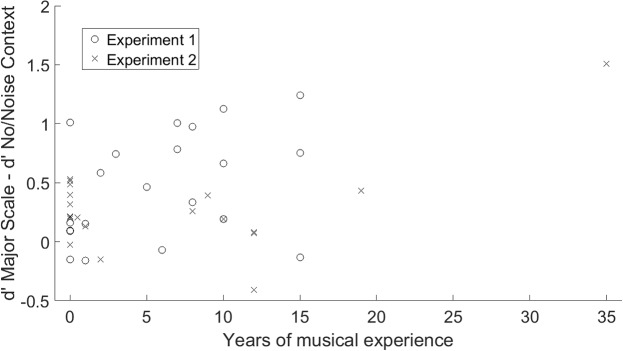
Benefit from familiar tonal context, measured as performance in the Major Scale condition minus performance in the No Context or Noise Context condition, as a function of years of musical experience. Data are plotted separately for Experiment 1 (melodic context, circles) and Experiment 2 (harmonic context, X’s).

**FIGURE 6:**
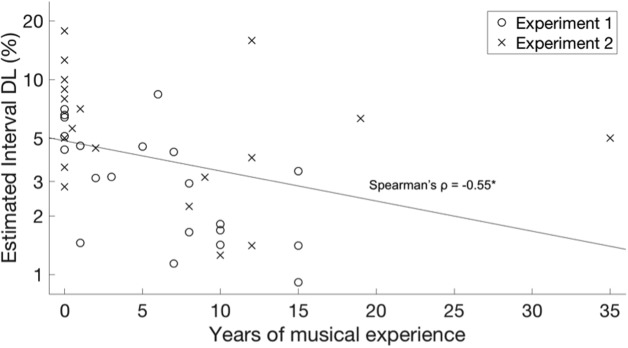
Difference limens (DLs) for interval discrimination in No Context or Noise Context conditions as a function of years of musical experience. Data are plotted separately for Experiment 1 (melodic context, circles) and Experiment 2 (harmonic context, X’s). The least-squares line across both experiments is plotted. A rank-order Spearman correlation was significant, *p* < 0.001.

A Spearman rank-order correlation revealed no significant relationship between years of musical experience and benefit over Major Scale relative to No Context, as shown in **Figure 5** (Spearman’s ρ = 0.17, *p* = 0.13). However, a significant negative correlation was observed between musical experience and baseline DL in the No Context condition, as shown in **Figure 6** (Spearman’s ρ = -0.55, *p* < 0.001).

One participant reported 35 years of musical training, and thus could be considered an outlier (across both experiments, the mean amount of musical training was 5.9 years, and the standard deviation was 7.2 years). For all results in Experiment 2, inclusion or exclusion of this outlier had no effect on any of the statistical conclusions.

### Discussion

The results of Experiment 2 were generally consistent with the hypothesis that establishing a familiar tonal context will improve the perception of pitch intervals; however, the observed benefit to performance was small in terms of changes in sensitivity index, *d′*, as in Experiment 1. In this experiment, the Major Scale context provided an advantage over all of the other context conditions except Whole-Tone Scale.

One of the goals of Experiment 2 was to discover whether a more strongly and unambiguously established tonal context would elicit a stronger effect. We did not find this result. The effect sizes from Experiment 2 are comparable to those from Experiment 1. Furthermore, in terms of the sensitivity index *d′*, the average benefit of the Major Scale context over No Context condition was somewhat smaller in Experiment 2 (0.28) than Experiment 1 (0.47). Although these effects are statistically significant, a benefit of less than 0.5 in terms of *d′* represents a relatively small real-world advantage, considering this index ranges from 0 at chance performance to greater than 2.5 at ceiling (for 90% hits and 10% false alarms, *d′* = 2.56).

Experiment 2 was also designed to determine whether the removal of tonal ambiguities present in the melodic context of Experiment 1 would lead to a more definite advantage for Major Scale context over Repetition context. This was confirmed by the results, as performance in the Major Scale context in Experiment 2 was significantly better than performance in Repetition context. Again, however, this benefit was rather small, averaging only 0.21 *d′* units when combined across the two standard interval sizes.

It is necessary to acknowledge two possible alternative explanations for the smaller-than-expected benefit of tonal context in Experiment 2, when comparing the results to those of Experiment 1. In Experiment 2, the context sequences were shorter than in Experiment 1, consisting only of two sounds rather than four. It is possible that reducing the length of the context sequence reduced the overall benefit from any context condition, which may be why the benefit even from repetition context was smaller in Experiment 2. This possibility could be further explored with a longer harmonic context sequence. It is also possible that the introduction of noise bursts in the Noise Context condition aided performance in this condition compared to the No Context condition in Experiment 1, leading to less room for improvement with tonal context. Adding noise bursts to the No Context condition in Experiment 1 could potentially resolve this question. Both of these alternative explanations represent limitations in the validity of comparing results from Experiment 1 to results from Experiment 2, and should be kept in mind when making this comparison.

## General Discussion

The present study investigated the effect of familiar tonal context on the perception of pitch intervals. We hypothesized that when discriminating between a small, in-tune interval and a large, mistuned interval, participants would perform better with an established (major) tonal context than with no context, or other less well-established contexts. The main results were that the Major Scale conditions provided an advantage over No Context in both experiments, over the Whole Tone condition in Experiment 1, and over the Repetition and Mistuned conditions in Experiment 2. An interaction in Experiment 1 revealed different results depending on standard interval size, with Major Scale only providing an advantage over No Context and Whole conditions for the two-semitone standard interval size.

Although we observed an effect of tonal context on interval perception, and a particular benefit for familiar tonal context, these conditions provided only a small benefit to performance over the No Context and Noise Context conditions, in terms of *d′*. These results suggest that learned tonal hierarchies may influence the accuracy of pitch interval perception, although the benefit may be slight in practical terms.

Using non-parametric Spearman correlation, we observed no significant correlation between the benefit of Major Scale context over No Context and years of musical experience. This outcome suggests that the benefit of familiar tonal context was not dependent on amount of musical training. In this respect, the outcomes are consistent with the conclusion of Bigand and Pineau (1997) that non-musicians are as sensitive to tonal structures as musicians, as well as more recent findings showing that when judging singers, even non-musicians are highly sensitive to mistunings from the equal-temperament scale (Hutchins et al., 2012; Larrouy-Maestri et al., 2015), presumably learned through passive exposure. It is also consistent with the finding that even non-musicians exhibit early right-anterior negativity (ERAN) in response to violations of tonal expectations (Koelsch et al., 2000). Importantly, however, this outcome does not imply that musicians and non-musicians were equally sensitive in general to pitch intervals, firstly as all scores are normalized by performance in the no/noise context condition, and secondly as all participants were tested at their individually estimated DL, which varied between participants. Indeed, the significant correlation between DLs and musical experience indicates that musically trained listeners had higher baseline performance on this task in the No Context condition.

It is worth noting that the task used in this study, in which participants compare a small, in-tune interval to a larger, mistuned interval, differs from that used in previous studies. The goal was to design a task that could in theory be strongly affected by tonal context. This task must be done based on the sizes of the intervals unless participants have access to tuning cues, which should in theory make the task much easier with tonal context. Another advantage of this task is that it allows for a shorter time between the end of the context sequence and the end of the test stimulus, also theoretically maximizing the effect of context. For these reasons, it seems likely that other tasks (removing the tuning cue, or traditional two-interval comparison) would likely find even smaller context effects. However, the conflation of tuning and interval size can be considered a limitation of the present study, in that performance may reflect access to tuning cues, precision of perceptual representation of interval size, or both. A task with no tuning cue (for example, with both intervals equally spaced around a “standard” common Western tonal interval) could be used to better resolve this question.

Our ability to discriminate very small changes in F0 on basic discrimination tasks is not mirrored in interval discrimination measured in isolation, where thresholds can be considerably higher (e.g., McDermott et al., 2010a). One possibility explored by the present study was that tonal context would play a much larger role in musical interval size discrimination. We had hypothesized that tonal hierarchies, by allowing listeners to hear successive pitches as points within a structure rather than as successive interval sizes, could provide a context in which performance for interval discrimination might better approximate performance for basic discrimination. In the present study, performance on interval discrimination was improved by the presence of a tonal context that suggested a familiar tonal hierarchy (major scale) to a greater degree than the improvement from the presence of incongruent tonal context. However, the degree of benefit was small, and was not sufficient to account for the differences in DLs between basic discrimination and interval discrimination, suggesting that even with tonal context, interval size perception is not as precise as basic pitch perception.

When cued using harmonic progressions, tonal context has robust effects on pitch processing as measured by response time (Bigand and Pineau, 1997; Tillmann et al., 2008). As discussed in those studies, the increase in response time in the presence of an unexpected or incongruous chord likely reflects cognitive priming, where the unexpected chord interferes with the detection of the target mistuning, rather than any enhancement of pitch representation through the tonal context in congruent conditions. The results from our study provide support for this interpretation: pitch interval discrimination is barely affected by congruent tonal contexts relative to priming by a single tone, and not affected at all in melodic context, where the benefit of each of these conditions relative to no context is dependent on standard interval size. One interpretation of our outcomes is that both harmonic and melodic context were effective in establishing a tonal hierarchy, but that tonal context provides only a modest benefit to pitch interval perception. Another possibility is that even the harmonic context sequences were not sufficiently long or harmonically rich to fully establish a musical key, such as the longer chord progressions used in previous studies.

The inclusion of control conditions in our study allowed us to further examine the source of the benefit from musical context to interval discrimination. In an earlier study of interval categorization and labeling in musicians (Wapnick et al., 1982), melodic context provided a large benefit over interval perception in isolation. However, our results suggest that some of this benefit may have merely been due to an effect of pitch reinforcement, as can be seen in the observed benefit from the Repetition context condition in Experiment 1 (melodic context). We can also conclude, however, that tonal congruence is a necessary component of effective tonal context, because context that established a familiar major-key tonality was superior to context conditions that failed to establish this tonality, such as the Mistuned Scale (in Experiment 2) and Whole-Tone Scale (in Experiment 1) contexts. For the harmonic (chord) contexts in Experiment 2, the tonally congruent context was also more effective than simple pitch reinforcement through repetition.

We measured interval discrimination thresholds at two standard interval sizes, and, consistent with previous findings, we found no significant effect of standard interval size on discrimination thresholds (Burns and Ward, 1978; McDermott et al., 2010a). However, standard interval size did have an effect on the pattern of results in the various context conditions in Experiment 1. Specifically, for a two-semitone standard interval size, the best performance was from the Major Scale condition, whereas for the five-semitone standard interval size, the best performance was from the Repetition condition. This difference may be driven in part by the different functions of the corresponding scale degrees in the major musical key where the reference tone is tonic. Both the major second (two semitones) and the perfect fourth (five semitones) are within the major key, but the perfect fourth is especially stable and closely related to tonic (Krumhansl and Kessler, 1982). The two next-largest intervals to these, against which participants would have to discriminate in the present study if Δ*F0* was near one semitone, also have different functions: neither the minor third (three semitones) nor the tritone (six semitones) belong to the major key, but the minor third is traditionally thought of as a consonant interval in Western music, while the tritone is thought of as dissonant.

Given the clear difference in tonal belongingness and consonance between the perfect fourth and the tritone, participants’ relatively poor performance in the five-semitone standard task – even with tonal context – is surprising. One relevant factor may be that all intervals in the present study were defined using the equal-temperament tuning system, wherein five semitones is defined as 2^(5/12)^. While some evidence suggests that a semitone defined in equal-temperament terms may be a perceptually relevant boundary for interval discrimination (Zarate et al., 2012), human ideals for musical intervals, whether measured by listener adjustment or musician production, do not agree perfectly with equal-temperament tuning, and this discrepancy may be even greater when measured within a musical context (Rakowski, 1990). Specifically, it appears that musical context may actually increase the deviation of this “ideal” from an equal-temperament standard for the ascending tritone. If this is the case in our experiment, participants may have greater difficulty discriminating an ascending perfect fourth from an ascending tritone (both defined by equal-temperament tuning) if their internal tuning allows for the tritone to be larger. In other words, it is possible that, with Δ*F0* generally less than one semitone, both the “small, in tune” and “large, mistuned” intervals in the five-semitone-standard task would fall into the “perfect fourth” category of musicians’ categorical interval perception, rather than straddling the boundary between the “perfect fourth” and “tritone” categories.

In summary, we explored the effect of familiar tonal context on a pitch interval discrimination task, a performance-based measure of pitch interval perception accuracy. In contrast to expectations of strong effects of tonal context on the accuracy of pitch-interval discrimination, we found a relatively small benefit. The results suggest that although tonal contexts can generate strong expectations, they do not produce substantial enhancements in the perceptual representations of pitch and pitch intervals.

## Author Contributions

JG and AO designed the experiments together. JG collected and analyzed the data. JG and AO wrote the manuscript.

## Conflict of Interest Statement

The authors declare that the research was conducted in the absence of any commercial or financial relationships that could be construed as a potential conflict of interest.
