# Supplementary material for: Familiar Tonal Context Improves Accuracy of Pitch Interval Perception
Source: Front Psychol. 2017 Oct 9;8:1753. doi: 10.3389/fpsyg.2017.01753 (PMC5640898; doi:10.3389/fpsyg.2017.01753)
Supplement: Supplementary file 1 [file Table_1.PDF]

Table S1. Benefit, in terms of d', of Major context over "No Context" (Exp 1) and "Noise Context" (Exp 2)

Experiment 1	2-semitone standard				5-semitone standard				
Subject number	Repetition	Mistuned	Whole Tone	Major	Repetition	Mistuned	Whole Tone	Major	Years of Musical Experience
1	0,46	-0,31	0,15	0,1	0,12	-0,67	0,03	-0,37	15
2	0,31	0,05	0,2	0,41	1,26	0,91	0,79	1,16	7
3	1,83	1,2	0,26	0,77	0,38	-0,17	-0,21	0,73	15
4	0,04	0,38	0,22	0,44	0,85	0,71	0,07	0,49	5
5	1,2	2,02	1,2	1,24	0,74	0,52	0,21	0,77	7
6	0,33	0,16	0,49	0,48	0,31	-0,05	-0,2	-0,1	10
7	0,5	0,59	0,77	1,57	1,34	0,76	0,32	0,38	8
8	0,49	0,4	-0,6	1,14	0,73	-0,5	-0,23	-0,47	8
9	0,89	0,47	0,85	1,27	0,94	0,12	1,85	0,98	10
10	1,15	-0,4	0,16	1	1,04	0,3	-0,18	0,33	10
11	1,39	1,9	1,63	2,37	-0,22	-0,37	-0,58	0,11	15
12	-0,33	0,28	0,01	0,11	-0,03	-0,04	-0,36	0,07	0
13	-0,63	-0,26	-0,57	-0,04	0,51	0,3	-0,34	-0,28	1
14	-0,02	0,21	0,47	0,78	0,11	-0,18	-0,45	-0,46	0
15	0,35	0,08	0,39	0,61	0,71	-0,2	-0,12	0,56	2
16	-0,06	0,43	0,16	0,27	0,1	0	0,18	-0,41	6
17	0,48	1,59	0,71	1,56	1,36	0,6	0,15	0,46	0
18	-0,89	-0,48	-0,33	-0,43	1,04	0,62	0,3	0,62	0
19	-0,09	0,68	0,23	1,28	0,23	0,13	0,68	0,21	3
20	0,18	0,11	-0,46	0,32	-0,35	-0,41	-0,26	-0,62	0
21	-0,04	0,22	0,35	0,07	0,07	-0,13	0,14	0,23	1

Experiment 2	2-semitone standard				5-semitone standard				
Subject number	Repetition	Mistuned	Whole Tone	Major	Repetition	Mistuned	Whole Tone	Major	Years of Musical Experience
1	-0,15	0,25	0,04	0,55	0,36	-0,15	0,05	-0,4	12
2	0,03	-0,1	0,14	0,33	-0,04	-0,26	0,22	0,45	9
3	0,36	1,44	0,4	0,72	-0,17	0,09	-0,23	0,15	19
4	-0,37	0,12	0,18	-0,37	0,5	-0,34	-0,21	0,53	12
5	-0,29	-0,58	-0,65	0,37	0,54	-0,92	-0,7	0,01	10
6	0,19	0,37	1,21	0,52	0,27	-0,5	-0,15	0	8
7	0,49	0,23	1,2	1,35	0,91	0,25	0,43	1,67	35
8	-0,54	0,02	-0,37	0,33	-0,89	-0,18	-0,86	-1,15	12
9	-0,46	-0,29	-0,24	-0,05	-0,25	-0,88	-0,39	0,44	0
10	0,16	-0,05	-0,21	0,46	0,89	0,52	0,63	0,6	0
11	-0,18	0	0,32	0,16	-0,1	0,15	0,22	0,1	1
12	-0,42	0,84	-0,18	-0,16	0,62	-0,12	0,31	0,59	0
13	0,1	-0,41	0,4	-0,05	0,2	-0,51	-0,26	-0,25	2
14	-0,27	-0,03	-0,06	-0,22	0,12	-0,05	0,26	0,64	0
15	-0,95	-0,36	-0,49	-0,32	0,64	0,25	1,26	1,11	0
16	0	-0,36	0,02	-0,12	0,11	0,24	-0,2	0,07	0
17	0,92	0,09	0,31	1,12	-0,6	-0,61	-0,16	-0,15	0
18	0,52	0,03	0,62	0,69	0,1	-0,17	-0,12	-0,05	0
19	0,71	0,01	0,46	0,77	-0,57	-0,47	0,3	0,27	0
20	-0,07	0,12	-0,05	0,09	0,44	0,37	0,47	0,32	0,5
